# An intrinsic temporal order of c-JUN N-terminal phosphorylation regulates its activity by orchestrating co-factor recruitment

**DOI:** 10.1038/s41467-022-33866-w

**Published:** 2022-10-17

**Authors:** Christopher A. Waudby, Saul Alvarez-Teijeiro, E. Josue Ruiz, Simon Suppinger, Nikos Pinotsis, Paul R. Brown, Axel Behrens, John Christodoulou, Anastasia Mylona

**Affiliations:** 1 Institute of Structural and Molecular Biology, University College London, London, UK; 2 School of Pharmacy, University College London, London, UK; 3 Institute of Structural and Molecular Biology, Birkbeck College, University of London, London, UK; 4 Cancer Stem Cell Laboratory, Institute of Cancer Research, London, UK; 5 Randall Division of Cell and Molecular Biophysics, Guy’s Campus, King’s College, London, UK; 6 Division of Cancer, Department of Surgery and Cancer, Imperial College, London, UK; 7 CR-UK Convergence Science Centre, Imperial College, London, SW7 2BU UK; 8 Present Address: Instituto de Investigación Sanitaria del Principado de Asturias (ISPA), Asturias, Spain; 9 Present Address: CIBERONC, Instituto de Salud Carlos III, Madrid, Spain; 10 Present Address: Friedrich Miescher Institute for Biomedical Research (FMI), Basel, Switzerland

**Keywords:** Kinases, NMR spectroscopy, Phosphorylation

## Abstract

Protein phosphorylation is a major regulatory mechanism of cellular signalling. The c-JUN proto-oncoprotein is phosphorylated at four residues within its transactivation domain (TAD) by the JNK family kinases, but the functional significance of c-JUN multisite phosphorylation has remained elusive. Here we show that c-JUN phosphorylation by JNK exhibits defined temporal kinetics, with serine63 and serine73 being phosphorylated more rapidly than threonine91 and threonine93. We identify the positioning of the phosphorylation sites relative to the kinase docking motif, and their primary sequence, as the main factors controlling phosphorylation kinetics. Functional analysis reveals three c-JUN phosphorylation states: unphosphorylated c-JUN recruits the MBD3 repressor, serine63/73 doubly-phosphorylated c-JUN binds to the TCF4 co-activator, whereas the fully phosphorylated form disfavours TCF4 binding attenuating JNK signalling. Thus, c-JUN phosphorylation encodes multiple functional states that drive a complex signalling response from a single JNK input.

## Abstract (web summary)

Phosphorylation at multiple sites is a major regulatory mechanism in cellular signalling. Here, the authors show that multisite phosphorylation of the c-JUN transcription factor by the JNK kinase exhibits intrinsic kinetics that allow a precise and timed regulation of the transcriptional output.

## Introduction

Protein phosphorylation is the most abundant and important post-translational protein modification^1,2^ and is a major regulatory mechanism of cellular signalling^3^. Deregulation of phosphorylation pathways commonly underlies disease aetiology^4^. Proteins often are phosphorylated not only at one but at multiple sites by a single kinase. Multisite phosphorylation is a key regulatory mechanism in signalling that controls many cellular processes, and has been suggested to provide a precise tool to generate complex functional outputs, often from a single kinase input^5–9^. This is often achieved via interactions between the multisite phosphorylated protein and partner proteins, and these phosphorylation-dependent interactions determine the biological output^10^. Transcription is an important biological process regulated by multisite phosphorylation^11,12^. We have previously provided a mechanistic understanding of the temporal dynamics of multisite phosphorylation of the Ternary Complex Factor (TCF) Elk-1 transcriptional coactivator by the ERK kinase^13^. Phosphorylation at eight S/T-P (Ser or Thr-Pro) sites of Elk-1 occurs with a defined kinetics, leading to intrinsic temporal phosphorylation events that effectively trigger and subsequently limit its transcriptional response by promoting and then inhibiting the recruitment of the Mediator complex^13^. Thus, understanding the intrinsic kinetics of multisite phosphorylation is key to gain insights on how collectively multiple phosphorylated sites by a kinase within a domain can shape the transcriptional response.

AP-1 (activator protein 1) activity is strongly induced in response to numerous signals including growth factors, cytokines and extracellular stress^14^. AP-1 consists of heterodimeric complexes formed by members of the JUN family (c-JUN, JUNB and JUND) together with the FOS proteins (c-FOS, FOSB, FRA1 and FRA2) and members of the ATF and CREB families. The proto-oncoprotein c-JUN is a major component of the AP-1 transcription factor that controls the expression of many genes important for cell proliferation and tumorigenesis^15,16^.

An important mechanism to stimulate AP-1 function is the N-terminal phosphorylation of the c-JUN transactivation domain (TAD) at multiple S/T-P sites by the c-JUN N-terminal kinase (JNK) group of mitogen-activated protein kinases (MAPK)^17^. The JNKs have three isoforms: JNK1, JNK2 and JNK3. JNK1 and JNK2 are expressed in almost all tissues, whereas the expression of JNK3 is restricted to neuronal tissues. Both JNK1 and JNK2 actively contribute to c-JUN phosphorylation, and are at least partly redundant^17^.

**Fig. 1:**
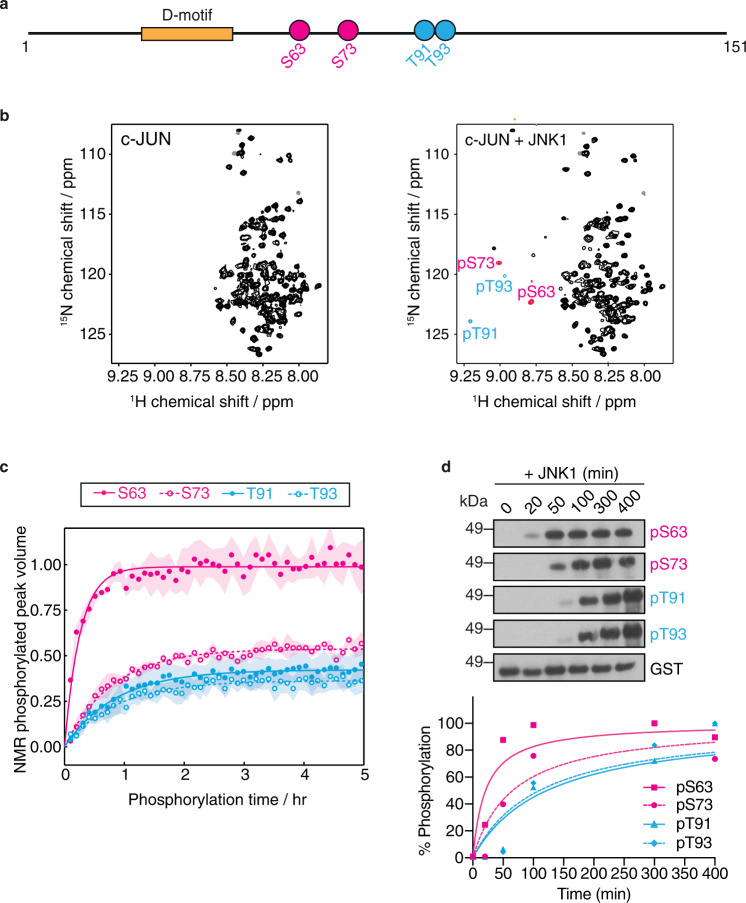
Multisite phosphorylation kinetics of the c-JUN TAD by JNK1. **a** Schematic representation of the c-JUN TAD sequence, showing the location of the fast (pink) and slow (cyan) S/T-P phosphorylation sites and the JNK binding motif (D-motif) (yellow). **b** 2D ^1^H,^15^N correlation spectra of (left) unphosphorylated c-JUN TAD, and (right) following 5 h in vitro phosphorylation by JNK1. Resonances from phosphorylated residues are highlighted with pink or cyan colouring corresponding to fast or slow sites respectively, with assignments as indicated. **c** Site-specific kinetics of c-JUN phosphorylation by JNK1 obtained from time-resolved NMR measurements, showing fits to single exponential build-ups. Data are plotted as the mean ± standard error of the mean (SEM) (*n* = 3 independent samples); error bars are presented as ribbons. **d** Western blot analysis of the in vitro phosphorylation kinetics of recombinant c-JUN TAD by JNK1 using phosphorylation-specific antibodies (top) and quantification of the detected protein levels at each time point using the Image Studio Lite Software (Licor) and normalized to total GST c-JUN TAD and subsequently to the maximum phosphorylation time point of each phosphorylation-specific antibody (bottom). The data are representative of a minimum of three biologically independent experiments (*n* = 3) performed with similar results. Source data are provided as a Source Data file.

JNKs phosphorylate the c-JUN TAD at four S/T-P residues, S63, S73, T91 and T93 (Fig. 1a), which modulates its interaction with various partner proteins to control target gene transcription^16,18–20^. The unphosphorylated c-JUN TAD interacts with MBD3, a subunit of the nucleosome remodelling and histone deacetylation (NuRD) repressor complex, which in turn recruits NuRD to AP-1 target genes to mediate gene repression. This repression is relieved by c-JUN phosphorylation following JNK activation^21^. Phosphorylated c-JUN at its TAD by JNK interacts with the TCF4 HMG-box transcription factor activating the *c-JUN* promoter^16^.

The multisite N-terminal phosphorylation of c-JUN by JNK is a key event linked to tumorigenesis. Mice in which the c-JUN phosphorylation sites S63 and S73 are mutated to alanines (*JunAA/JunAA*) develop normally but show specific defects in oncogenic transformation^22^. Moreover, in the intestine both MBD3 and TCF4/*b*-catenin control cellular proliferation and tumorigenesis via JNK/c-JUN N-terminal phosphorylation. Mice lacking MBD3 (*mbd3*^ΔG/ΔG^) show increased intestinal progenitor proliferation, which can be rescued upon deletion of one *c-jun* allele (*mbd3*^ΔG/ΔG^; *c-jun*^ΔG/+^)^21^. In a mouse model of intestinal cancer that is heterozygous for a nonsense mutation at codon 850 of the *Apc* gene (*Apc*^Min/+^), mice that are crossed to *JunAA* mice (*Apc*^Min/+^; *JunAA/JunAA*) exhibit greatly reduced intestinal tumors comparing to the *Apc*^Min/+^ mice^16^. However, although the biological significance of JNK c-JUN S63 and S73 N-terminal phosphorylation and its role in tumour development have been clearly demonstrated, the roles of the four individual phosphorylation sites and the interplay among them, and particularly how they might cooperate to regulate the temporal cofactor recruitment and thus transcriptional output, have not yet been explored.

In this study, we show that c-JUN N-terminal multisite phosphorylation by JNK occurs with a range of rates, leading to intrinsic temporal phosphorylation states, both in vitro and in vivo. This differential kinetic behaviour arises from differences in the primary sequence of the phosphorylation sites, as well as their position relative to the kinase docking motif. Finally, we examine the functional impact of the differential kinetics of c-JUN N-terminal phosphorylation and identify that c-JUN TAD exhibits three principal temporal phosphorylation states: an unphosphorylated repressive state that binds the MBD3 repressor, a double phosphorylated transcriptionally active state that binds the TCF4 transcriptional activator, and finally a fully phosphorylated inactive form that disfavours TCF4 binding and thus attenuates JNK signalling. Our results, therefore, demonstrate that the c-JUN N-terminal phosphorylation is a sequential phosphorylation cascade that controls in a time-dependent manner recruitment of c-JUN cofactors, thereby auto adjusting its transcriptional output via a single JNK kinase signalling input.

## Results

### Kinetics of c-JUN N-terminal phosphorylation by JNK

As a first step to explore the phosphorylation kinetics of the c-JUN TAD by JNK, we used time-resolved NMR spectroscopy to monitor the modification of individual c-JUN phosphosites in real time^23–25^. We expressed and purified c-JUN TAD (residues 1-151) containing all four phosphorylation sites, S63, S73, T91 and T93 and the MAPK binding motif (D-motif, residues 32-50) which controls phosphorylation of these sites^26,27^ (Fig. 1a). The narrow chemical shift dispersion in the 2D ^1^H,^15^N correlation spectrum of unphosphorylated c-JUN TAD (Fig. 1b) indicates that the domain is intrinsically disordered, and following backbone resonance assignment (Supplementary Fig. 1a), an analysis of secondary chemical shifts^28^ showed no significant populations of secondary structure (Supplementary Fig. 1b), consistent with previous reports of c-JUN fragments comprising residues 1-123 and 1-276^29^.

We reacted ^15^N-labelled c-JUN TAD with recombinant active JNK1 and performed time-resolved NMR to monitor its modification rate in vitro (Fig. 1b and Supplementary Fig. 2). Due to the change in their chemical environment, phosphorylated serine and threonine (pS and pT) amide resonances appear in a distinctive and well-resolved region of the 2D ^1^H,^15^N correlation spectrum^30^, and their intensities can therefore be readily tracked as a function of time (Fig. 1c). pS and pT resonances that were observed to build up over time were assigned to the four phosphorylation sites via sequential site-directed mutagenesis to alanine (i.e. S63A, S73A, T91A and T93A) to eliminate phosphorylation (Supplementary Fig. 3). The narrow chemical shift dispersion observed throughout the phosphorylation reaction (Fig. 1b and Supplementary Fig. 2) indicates that the c-JUN TAD domain remains disordered in all its phosphorylation states, consistent with previous analyses of hyperphosphorylated forms of c-JUN 1-123 and 1-276 by ERK2^29^.

Following addition of JNK1, the build-up of phosphorylated resonances displayed a range of rates, in the order S63 > S73 > T91 ≈ T93 (Fig. 1c). Similar observations were made by reacting c-JUN TAD with recombinant active JNK2 (Supplementary Fig. 4a). Phosphorylation of all residues followed exponential kinetics with no discernible lag phases, which provided early evidence that there is no strong cooperativity between adjacent phosphorylation sites. This is explored in further detail below. The kinetics observed by NMR were confirmed by immunoblotting of the in vitro reactions with phosphorylation-specific antibodies against each of the phosphorylated residues (Fig. 1d). The site specificity of these phospho-antibodies was verified using alanine mutants for each phosphorylation site (Supplementary Fig. 4b).

**Fig. 2:**
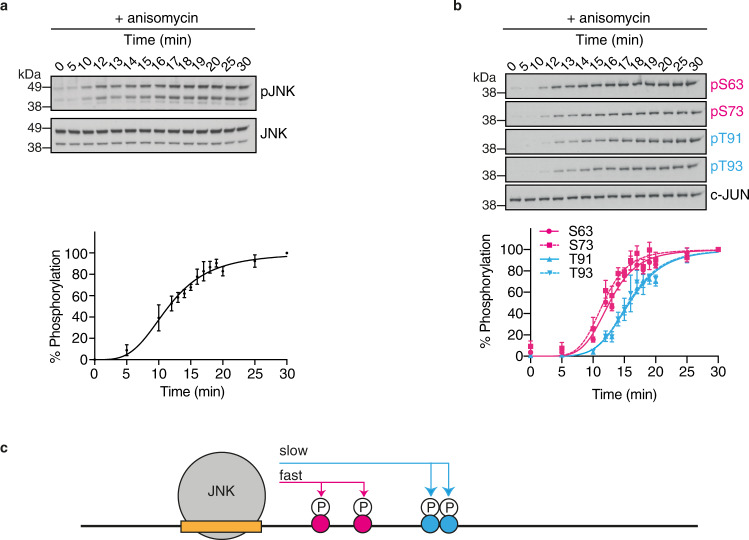
Kinetics of the c-JUN N-terminal phosphorylation in vivo. **a** Representative Western blot analysis of the phosphorylation (activation) of endogenous JNK in NIH3T3 cells following anisomycin treatment (top), and quantification of the detected protein levels using the Image Studio Lite Software (Licor) and normalized to total JNK. Graphs show the mean ± standard error of the mean (SEM) (*n* = 3 biologically independent experiments) (bottom). **b** Representative western blot analysis of the phosphorylation kinetics of endogenous c-JUN in NIH3T3 cells following anisomycin treatment using phosphorylation-specific antibodies (top), and quantification of the detected protein levels using the Image Studio Lite Software (Licor) and normalized to total c-JUN and subsequently to the maximum phosphorylation time point of each phosphorylation-specific antibody. Graphs show the mean ± standard error of the mean (SEM) (*n* = 3 biologically independent experiments) (bottom). Source data are provided as a Source Data file. **c** Schematic summary of the observed phosphorylation kinetics of the c-JUN N-terminal phosphorylation by JNK.

To examine the N-terminal phosphorylation kinetics of c-JUN in vivo, we used anisomycin treatment to stimulate JNK phosphorylation and activation. This occurred with a half-time of about 12 min following a ca. 5 min lag period, and then remained in its active phosphorylated form for the 30 min duration of measurements (Fig. 2a). We then monitored the phosphorylation rates of S63, S73, T91 and T93 within endogenous c-JUN by immunoblotting (Fig. 2b). Consistent with our in vitro measurements, phosphorylation of S63 and S73 occurred rapidly following JNK activation, with half-times of ca. 12 min, while phosphorylation of T91 and T93 occurred more slowly, with a half-time of ca. 15–17 min (Fig. 2b). Back-dilution of in vitro phosphorylated recombinant GST c-JUN TAD protein and protein extracts from anisomycin-treated cells confirmed that the signal intensity of each of the phospho-antibodies was proportional to sample amount analysed, ensuring that antibody signals were within the linear range of measurements (Supplementary Fig. 4c, d). Taken together, these data indicate that phosphorylation of the c-JUN TAD proceeds under physiological conditions through two phases, in which S63 and S73 are phosphorylated more rapidly followed by T91 and T93 (Fig. 2c).

### JNK1 operates independently on individual c-JUN phosphorylation sites

**Fig. 3:**
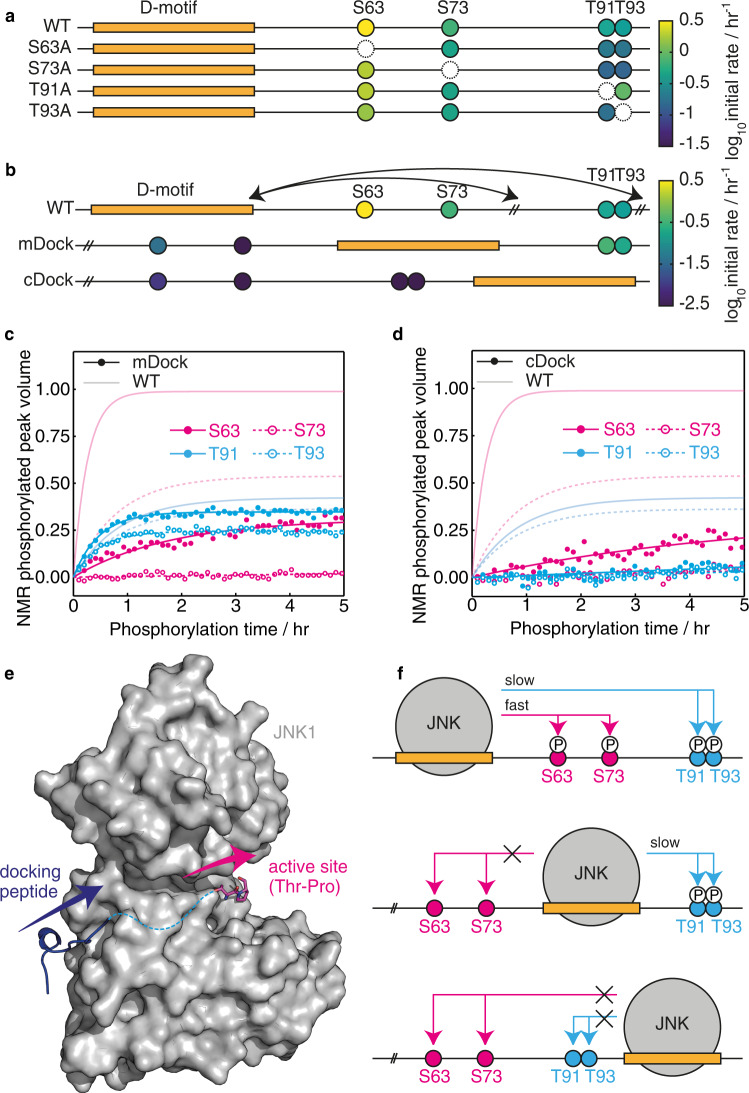
Effect of relative distance and orientation of the S/T-P sites from the D-motif on the c-JUN TAD N-terminal progressive multisite phosphorylation. **a**, **b** Schematic representation of the c-JUN TAD constructs S63A, S73A, T91A and T93A (**a**), and mDock and cDock (**b**) comparing to wild-type (WT). The initial rate of phosphorylation of residues by JNK1, determined by time-resolved NMR spectroscopy, is indicated according to the displayed colour scale. **c**, **d** Site-specific build-up curves of the phosphorylation of c-JUN mDock (**c**) and cDock (**d**) variants by JNK1, showing the mean of two biologically independent samples and fitted to single exponential build-up curves; pale lines show fits of wild-type c-JUN TAD (Fig. 1c) for comparison. Source data are provided as a Source Data file. **e** Crystal structure of JNK1 (grey surface) bound to a D-motif peptide (dark blue), superimposed with a crystal structure of a consensus T-P substrate peptide (magenta) bound to the homologous DYRK kinase (not shown). Arrows indicate the orientation of the bound peptides, with a hypothetical linker sequence indicated (dashed cyan). **f** Schematic representation summarizing the effect of the relative distance and orientation of the c-JUN TAD S/T-P residues from the D-motif on the temporal dynamics of the c-JUN N-terminal phosphorylation.

Having identified variations in the phosphorylation rates of c-JUN TAD residues, we sought to understand their molecular basis. To do this, we first examined whether any cooperativity existed in the phosphorylation of different residues. As noted above, the phosphorylation kinetics of individual residues could be fitted closely by exponential functions, without any evidence of lag phases (Fig. 1c), which indicated that phosphorylation of a particular site was not a pre-requisite for phosphorylation of another site. This conclusion is further supported by analysis of the phosphorylation kinetics of the single-site alanine variants, S63A, S73A, T91A and T93A, using time-resolved NMR. Alanine replacement of any of the individual sites did not result in substantial changes in phosphorylation rates of the other phosphorylated sites (Fig. 3a and Supplementary Fig. 5a).

### Location of phosphorylation sites determines phosphorylation efficiency

To further understand the factors underlying the variation in c-JUN phosphorylation rates by JNK1, we hypothesised that differences in kinetics may reflect the varying spatial proximity of phosphorylation sites relative to the D-motif, particularly given that the fast S63 and S73 sites are located closer to the D-motif than the slower T91 and T93 sites. To test this, we engineered two c-JUN TAD constructs, mDock and cDock, in which the position of the D-motif was transposed relative to the fast and slow groups of phosphorylation sites (Fig. 3b and Supplementary Fig. 5b).

We measured the phosphorylation kinetics of mDock and cDock variants using time-resolved NMR (Fig. 3b–d and Supplementary Fig. 3). The increased proximity of T91 and T93 in the mDock variant did not result in more rapid phosphorylation, but instead their phosphorylation rates remained very similar to those in the wild-type TAD construct (Fig. 3c). However, the phosphorylation of sites N-terminal to the D-motif was strongly suppressed, with only very slow modification of the normally fast S63 site observed in both mDock and cDock variants (Fig. 3c, d). These observations can be rationalised by consideration of the three-dimensional structural model of JNK1, showing a bound D-motif peptide^31^ and a consensus T-P substrate peptide (generated by alignment of a crystal structure of DYRK)^32^ (Fig. 3e). These peptides bind to the enzyme in a common orientation, with only a short distance from the C-terminal of the D-motif to the N-terminal of the phosphorylation site, such that phosphorylation sites C-terminal to the D-motif can readily access the active site. In contrast, phosphorylation sites N-terminal to the D-motif require the intervening linker (if long enough) to fold back upon itself at a high entropic cost, resulting in a significantly slower rate of phosphorylation. Taken together, these data indicate that although the relative distance of the phosphorylation sites to the D-motif is not essential in defining the temporal order of c-JUN multisite phosphorylation by JNK1, efficient phosphorylation does require positioning of the phosphorylation sites downstream of the D-motif (Fig. 3f).

### Primary amino acid sequence governs phosphorylation rate

**Fig. 4:**
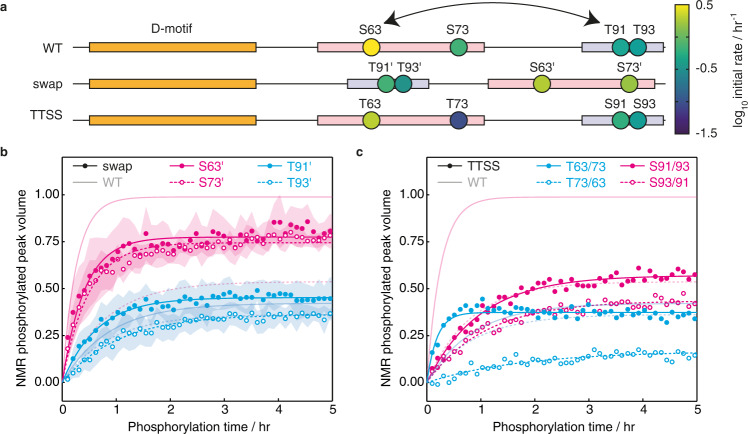
Effect of primary sequence of the S/T-P sites on the c-JUN TAD N-terminal phosphorylation kinetics. **a** Schematic representation of the c-JUN TAD constructs swap and TTSS comparing to wild-type (WT). The initial rate of phosphorylation of residues by JNK1, determined by time-resolved NMR spectroscopy, is indicated according to the displayed colour scale. **b** Site-specific build-up curves of the phosphorylation of c-JUN swap variant by JNK1. Data are plotted as the mean ± standard error of the mean (SEM) (*n* = 3 biologically independent samples) and fitted to single exponential build-up curves; error bars are presented as ribbons and pale lines show fits to wild-type c-JUN TAD (Fig. 1c) for comparison. **c** Site-specific build-up curves of the phosphorylation of c-JUN TTSS variant by JNK1. Data are plotted as the mean of two biologically independent samples and fitted to single exponential build-up curves; pale lines show fits to wild-type c-JUN TAD (Fig. 1c) for comparison. Source data are provided as a Source Data file.

Next, we sought to differentiate the effects of the primary sequence of c-JUN and the distance of phospho-sites from the D-motif on the rate of phosphorylation. For this purpose, we engineered a ‘swap’ variant, in which a sequence containing the fast S63 and S73 phospho-sites was exchanged with a sequence containing the slower T91 and T93 sites (Fig. 4a and Supplementary Fig. 5c), and measured the phosphorylation rates of individual sites by time-resolved NMR (Fig. 4a, b and Supplementary Fig. 3). We observed that the rate of S63 phosphorylation slightly decreased, which may be consistent with a decrease in its effective concentration given the longer linker length^33^. However, both residues, S63 and S73, despite now being further away from the D-motif than T91 and T93, were still phosphorylated with higher rates than T91 and T93, whose modification rate remained at similar levels than the wild-type (Fig. 4a, b).

To investigate whether the effect of primary sequence on phosphorylation could be explained by residue type (serine versus threonine) alone, we generated a c-JUN TAD variant in which serine and threonine residues were exchanged (‘TTSS’ variant: S63T/S73T/T91S/T93S), and measured phosphorylation rates by time-resolved NMR (Fig. 4a, c and Supplementary Fig. 5c). The TTSS mutant showed substantial changes in the phosphorylation kinetics, such that the final ordering of phosphorylation rates was T > S ≈ S > T, highlighting the importance of the identity of the individual amino acid being phosphorylated (Fig. 4a, c). The observations above were further corroborated by the fact that in the 2SA mutant (S63A/S73A), T91 and T93 are still phosphorylated with significantly decreased rates when compared to the rates of S63 and S73 in the wild-type, despite being the only substrates of the kinase in this case (Supplementary Figs. 3, 5a). Taken together, these data therefore suggest that both the preference of the kinase for the serine over the threonine residues, and the primary sequences surrounding the S-P and the T-P sites, contribute to determine the kinetics of the c-JUN N-terminal phosphorylation by JNK1.

### c-JUN phosphorylation kinetics regulates co-factor binding

**Fig. 5:**
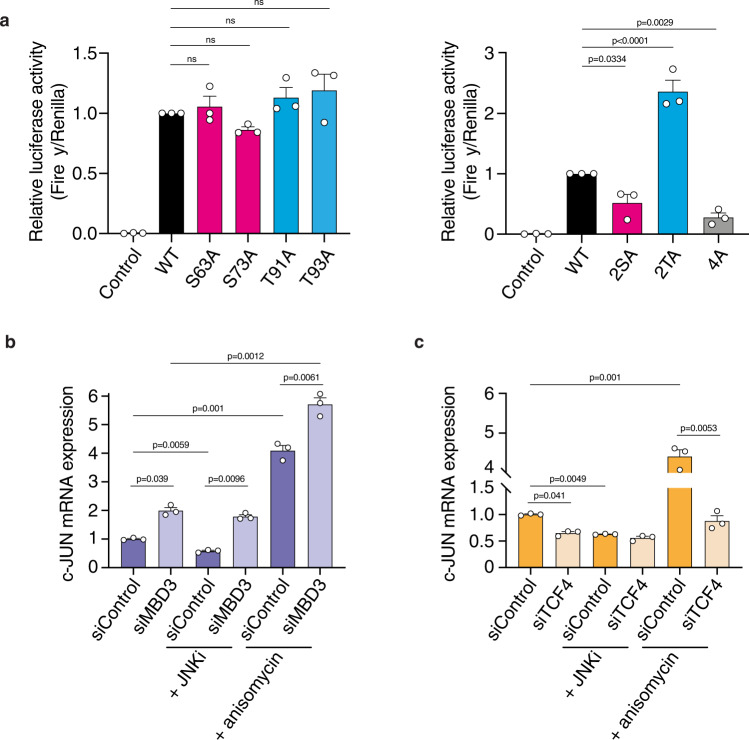
Effect of c-JUN phosphorylation on transcriptional activity. **a** NIH3T3 cells were transiently transfected to express: empty vector as a control, or c-JUN WT or c-JUN alanine mutants, firefly luciferase reporter gene, tk-Renilla as transfection control, and constitutively active MEKK as an upstream activator of c-JUN phosphorylation. Firefly luciferase activity was measured and normalized to Renilla luciferase activity and shown relative to c-JUN WT. Data are shown as the mean ± standard error of the mean (SEM). Statistical significance was determined by One-way ANOVA with Dunnett’s multiple comparisons test (*n* = 3 biologically independent experiments). Non-significant terms are indicated as ‘ns’. **b**, **c** Graphs showing the difference in c-JUN mRNA expression between control, siMBD3 (**b**) or siTCF4 (**c**) transfected HCT116 human colorectal cancer cells. HCT116 cells were further treated with JNKi or anisomycin. Data are shown as the mean ± standard error of the mean (SEM). Statistical significance was determined by One-way ANOVA with Tukey’s multiple comparisons test (*n* = 3 biologically independent experiments). Source data are provided as a Source Data file.

Having identified groups of fast (S63, S73) and slow (T91, T93) phosphorylation sites, we next explored the biological function of the kinetics of the c-JUN phosphorylation by testing the effect of each phosphorylation kinetic group of the c-JUN TAD on its ability to activate transcription. For that purpose, we first analysed the effect of each individual c-JUN phosphorylation site with a *c-jun* luciferase reporter gene assay using constitutively active MEKK as an upstream activator of c-JUN phosphorylation (pFC-MEKK), which specifically activated JNK and not the other MAPK kinases (p38 or ERK) (Supplementary Fig. 6a, b). Individual single c-JUN alanine point mutants (S63A, S73A, T91A, T93A) had no significant effect on the reporter activity (Fig. 5a). However, a c-JUN mutant bearing alanine mutations in both S63 and S73 sites (2SA) greatly decreased the reporter activity when compared to the wild-type protein, which is consistent with previous observations^34^. Strikingly, alanine substitution of both the slower T91 and T93 sites (2TA) exhibited increased reporter activity comparing to the wild-type. Lastly, alanine substitution of all four phosphorylation sites (4A) showed decreased reporter activity relative to wild-type protein, similar to that seen with the 2SA variant (Fig. 5a). Taken together, these data suggest that the two phosphorylation kinetic groups of the c-JUN TAD have distinct activating and inhibitory effects on c-JUN transcriptional activity.

To investigate the role of the transcriptional repressor MBD3 and the co-activator TCF4 in regulating c-JUN activity, we knocked-down *MBD3* or *TCF4* expression with small interfering RNAs (siRNAs) (Supplementary Fig. 6c, d). c-JUN autoregulates its own promoter^35^ and thus c-JUN activity in response to JNK inhibition/activation was assessed using *c-JUN* expression. As expected, pharmacological JNK inhibition decreased endogenous *c-JUN* mRNA levels, but anisomycin treatment increased *c-JUN* mRNA levels (Fig. 5b, c). siRNA-mediated depletion of *MBD3* significantly enhanced basal *c-JUN* mRNA levels and *c-JUN* mRNA levels were further increased by JNK activation (Fig. 5b). Conversely, *MBD3* depletion abrogated repression of *c-JUN* by JNK inhibition, suggesting that MBD3 is required for *c-JUN* repression in the absence of JNK activity (Fig. 5b).

*TCF4* depletion resulted in a modest decrease in *c-JUN* mRNA levels, which was unaltered by JNK inhibition. In contrast, *TCF4* depletion largely abolished c-JUN transcriptional activation by anisomycin (Fig. 5c) indicating that TCF4 contributes to the increased c-JUN activity induced by N-terminal phosphorylation.

**Fig. 6:**
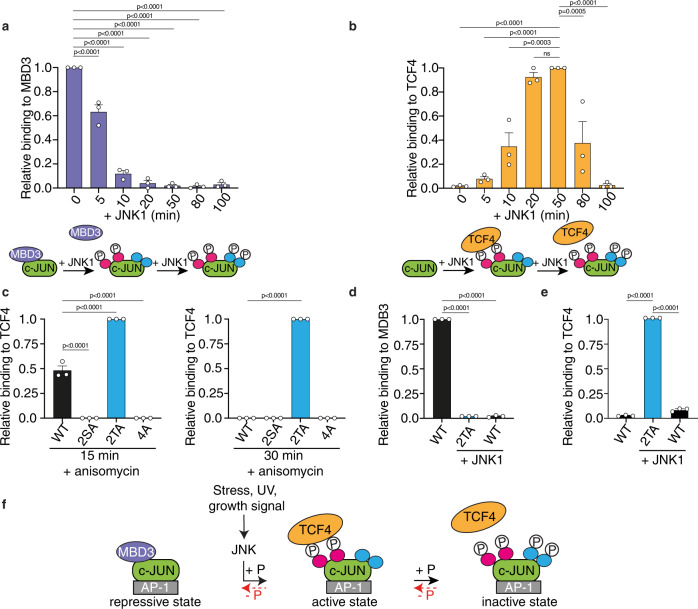
Effect of c-JUN temporal phosphorylation events on protein partners binding. **a**, **b** HCT116 cell extracts were used in pull-down assays, using GST-c-JUN TAD as bait, phosphorylated by JNK1 at the indicated times. Recovered proteins were analysed by immunoblotting for MBD3 (**a**) or TCF4 (**b**). Quantification of the detected protein levels was performed using the Image Studio Lite Software (Licor), and normalized to the bound GST-c-JUN TAD and relative to the 0 min (**a**) or 50 min (**b**) prey binding (top). Schematic representation of the recovery of MBD3 (**a**) and TCF4 (**b**) from the performed pull-downs (bottom). **c** The indicated flag-tagged c-JUN wild-type or derivatives, transiently overexpressed in HCT116 cells, treated with either JNKi or anisomycin for the indicated time, were immunoprecipitated using Flag antibody. Immunoprecipitates were analysed for interaction with TCF4 and the quantification of the detected TCF4 protein levels that were immunoprecipitated was performed using the Image Studio Lite Software (Licor), and normalized to the immunoprecipitated Flag-c-JUN and relative to 2TA + anisomycin. **d**, **e** Recombinant unphosphorylated or fully phosphorylated GST wild-type c-JUN TAD or GST 2TA TAD derivative were used as bait in pull-downs with recombinant MBD3 (**d**) and TCF4 (**e**). Recovered proteins were analysed by immunoblotting for MBD3 (**d**) and TCF4 (**e**). Quantification of the detected protein levels was performed using the Image Studio Lite Software (Licor), and normalized to the bound GST-c-JUN TAD and relative to the maximum unphosphorylated WT (**d**) or 2TA + JNK1 (**e**). Data are shown as the mean ± standard error of the mean (SEM). Statistical significance was determined by One-way ANOVA with Dunnett’s multiple comparisons test (*n* = 3 biologically independent experiments). Non-significant terms are indicated as ‘ns’. Source data are provided as a Source Data file. (**a**, **b**, **c**, **d** & **e**). **f** Schematic summary model of the biological function of c-JUN N-terminal progressive phosphorylation by JNK.

To gain further mechanistic insight on how the intrinsic kinetics of c-JUN N-terminal phosphorylation differentially control its transcriptional activity, we investigated the relationship between c-JUN phosphorylation and its interactions with the transcriptional repressor MBD3 and the activator TCF4. We conducted glutathione S-transferase (GST) pull-down experiments using recombinant GST-c-JUN TAD phosphorylated for different times such that phosphorylation was either mainly at S63 and S73 or complete (Fig. 1d), and tested the recovery of endogenous MBD3 and TCF4 respectively from HCT116 cell extracts. In this assay, MBD3 interacted exclusively with unphosphorylated c-JUN TAD, and binding was greatly reduced as soon as c-JUN TAD phosphorylation was initiated and abolished by the time the fast sites were significantly phosphorylated (Fig. 6a and Supplementary Fig. 6e). Interaction with TCF4 was dependent on c-JUN TAD phosphorylation, and binding coincided with phosphorylation of S63 and S73. c-JUN interaction was progressively reduced as the T91 and T93 phosphorylation increased and abolished at the latest time point when c-JUN was fully phosphorylated at all four sites (Fig. 6b and Supplementary Fig. 6f).

To examine the contribution of the different kinetic phosphorylation groups of c-JUN TAD to the TCF4/phosphorylated c-JUN interaction, we performed co-immunoprecipitation assays using HCT116 cells, transiently expressing Flag-tagged c-JUN or mutants (2SA, 2TA and 4A), to recover binding of endogenous TCF4 from cell extracts treated either with JNK inhibitor (JNKi) or with anisomycin to induce partial or complete c-JUN phosphorylation (Fig. 6c and Supplementary Fig. 7a). JNK phosphorylation and c-JUN phosphorylation of the individual S/T-P residues exhibited the same kinetics with anisomycin treatment in HCT116, as seen in NIH3T3 cells (Supplementary Fig. 7b, c). Consistent with pull-down experiments, the interaction of TCF4 with c-JUN coincided with partial phosphorylation of c-JUN, and it was abolished when c-JUN was fully phosphorylated (Fig. 6c and Supplementary Fig. 7d–f). This assay also showed that TCF4 binding is dependent on phosphorylation of S63 and S73, since their alanine substitution in both the 2SA and 4A c-JUN mutants did not allow recovery of TCF4. TCF4/c-JUN interaction antagonised by T91/T93 phosphorylation, as mutation of these residues enhanced TCF4/c-JUN interaction and prevented TCF4 release (Fig. 6c and Supplementary Fig. 7d–f).

Next, we sought to understand whether the c-JUN phosphorylation states impact directly on cofactor binding. For that purpose, we expressed recombinant MBD3 and TCF4 in *E. Coli*, and performed glutathione S-transferase (GST) pull-down experiments using recombinant unphosphorylated or fully phosphorylated wild-type GST-c-JUN TAD and phosphorylated 2TA mutant, and tested the recovery of recombinant MBD3 and TCF4 respectively. Recombinant MBD3 bound to unphosphorylated c-JUN TAD and this interaction was abrogated by phosphorylation (Fig. 6d and Supplementary Fig. 7g). Recombinant TCF4 binding to the c-JUN TAD was dependent on c-JUN S63/S73 phosphorylation as it showed increased binding to the 2TA mutant, which was greatly reduced on the fully phosphorylated wild-type c-JUN TAD (Fig. 6e and Supplementary Fig. 7h), demostrating that T91/T93 phosphorylation antagonises binding of TCF4 in a direct manner. Thus c-JUN N-terminal phosphorylation directly controls the interaction with MBD3 and TCF4, respectively.

## Discussion

In this study we have explored the multisite phosphorylation of the c-JUN TAD by JNK, both in vivo and in vitro, and have identified a hierarchy of phosphorylation rates that leads to an intrinsic temporal signalling response. We have shown that c-JUN N-terminal phosphorylation is associated with three distinct functional phosphorylated states. Unphosphorylated c-JUN remains transcriptionally repressed via its direct binding to the MBD3 subunit of the NurD repressor complex. Rapid phosphorylation of S63/S73 leads to an active state in which the MBD3 repressor is displaced from the repressive, unphosphorylated state, allowing direct binding of the transcriptional activator TCF4, thus triggering c-JUN transcriptional activity. Subsequently, slower phosphorylation of T91/T93 restores c-JUN to a transcriptionally inactive state in which TCF4 binding becomes disfavoured. This behaviour thus provides a self-limiting safety mechanism to attenuate JNK signalling, independent of the engagement of phosphatases that must ultimately restore c-JUN to its repressive, unphosphorylated state (Fig. 6f).

The c-JUN TAD is now the second example of a transcription factor, after the TCF Elk-1^13^, for which an intrinsic hierarchy of phosphorylation rates has been shown to orchestrate the engagement of protein partners, leading to the autoregulation of a complex biological response elicited by a linear input signal. The existence of intrinsic functional kinetics of phosphorylation sites within multisite phosphorylated transcription factors thus appears to be a more general phenomenon, at least within the MAPK signalling pathway. For both c-JUN and Elk-1, phosphorylation kinetics convert the initial kinase signal into a pulse of transcriptional activity, however using different mechanisms. We have previously shown that the temporal kinetics of multisite phosphorylation of Elk-1 TAD controls the interaction with a single protein, the MED23 subunit of the Mediator complex, to both positively and negatively regulate the transcriptional activity^13^. In contrast, the multisite phosphorylation of c-JUN orchestrates the coordinated recruitment and displacement of co-factor proteins, the combination of which determines the biological response. The response of c-JUN to phosphorylation is therefore potentially more complex, as the signalling output is dependent on interactions with several protein partners. For example, c-JUN phosphorylation is essential for intestinal tumor formation in epithelial cells via the MBD3 and TCF4 interactions^16,21^ yet triggers apoptosis in neuronal cells via its interaction with Bag1-L co-activator^19,36,37^. It appears likely that such a mechanism would allow binding of differential readers, e.g. tissue specific repressors and activators, of the c-JUN N-terminal phosphorylation kinetics to elicit distinct biological responses in different tissue types.

We have found that JNK kinase exhibits a strong preference for the primary amino acid sequences of the S63 and S73 (Fig. 4). The distance between the kinase binding site and the phosphorylation site, which modulates the effective concentration of the phosphorylation site^38^, did not strongly influence the observed phosphorylation kinetics. However, the relative orientation of docking and phosphorylation sites was critical, indicating that steric accessibility remains a significant factor. A sterically favoured positioning downstream of the D-motif rather than upstream, similarly to what we have observed for c-JUN TAD, has also been shown to be required for efficient phosphorylation of substrate sites by the MAP kinase ERK^39,40^.

A comparison of the mechanisms by which the MAP kinases JNK1 and ERK2 recognise their substrate phosphorylation sites on c-JUN and Elk-1 TAD respectively, to generate the kinetics of multisite phosphorylation shows significant differences. The similar phosphorylation rates observed for residues in different c-JUN alanine variants provide insight into the mechanism of multisite phosphorylation by JNK1, indicating that the JNK1 active site cannot be completely saturated by the c-JUN phospho-sites, as the elimination of one phosphorylation site does not lead to a redistribution to other sites. This appears to contrast with the competitive mechanism of multisite phosphorylation observed in Elk-1, in which its active site is saturated by substrates such that elimination of a certain kinetic group of phospho-sites results in a redistribution of the kinase to the remaining sites and hence more rapid phosphorylation^13^. This competitive model of Elk-1 multisite phosphorylation is governed by the position of individual Elk-1 substrate sites relative to the kinase docking interactions, and does not allow substrate site preference by the kinase, unlike what we observe for c-JUN. These differences could reflect the fact that Elk-1 is targeted by different MAPK kinases (ERK, p38 and JNK) each generating distinct phosphorylation kinetics^13^. Such a competitive mechanism appears more flexible, allowing for different phosphorylation patterns depending on the kinase. In the case of c-JUN, which is exclusively phosphorylated by JNK family kinases, substrate site preference appears to be a more conservative mechanism that would not only ensure accurate timing of transcriptional activation and subsequent elimination but potentially filter binding of other MAPK kinases^41^.

The identification of three functional states associated with c-JUN N-terminal phosphorylation may have implications for the development of novel therapeutic cancer strategies. The JNK signalling pathway has been a desirable drug target for years, but so far JNK inhibitors have not been translated into clinical use. The main reason is the lack of specificity of the current inhibitors and cellular toxicity^42,43^. JNK plays an important role in various normal biological functions and its direct inhibition could have undesired side effects. Inhibitors targeting specific JNK-mediated downstream substrates and cellular events, such as phospho-c-JUN or the interaction of phospho-c-JUN binding partners, may show increased tumour specificity and efficacy. Our results suggest that in the case of c-JUN, the active doubly phosphorylated pS63/pS73 c-JUN form, and its interaction with activators, would be the most promising candidates for more personalised therapeutic intervention.

In summary, in this study we have shown that c-JUN multisite phosphorylation by JNK exhibits an intrinsic kinetics that leads to a precise and timed regulation of the transcriptional output of the JNK signalling pathway, where both positive and negative outputs are generated by a single kinase input. The concept of the temporal phosphorylation pattern and its translation to a self-eliminating transcriptional mechanism thus appear to be more general in the MAPK signalling. This mechanism contrasts the dogma of the classic ‘on and off switch’ of the transcriptional response via the dual role of kinases and phosphatases in MAPK signalling and perhaps more general in signalling pathways, revealing that the temporal dimension of multisite phosphorylation of a protein domain allows for more elaborate signalling responses. We speculate that the intrinsic kinetics of post-translational modifications across multiple sites might provide a simple and universal mechanism for the generation of a complex temporal biological response to a kinase signalling event.

## Methods

### Molecular techniques

Plasmid constructions, protein analysis by SDS-PAGE and immunoblotting used standard methods. c-JUN point mutations were generated by PCR-based mutagenesis using the site-directed mutagenesis kit (New England Biolabs). cDock, mDock and swap c-JUN, MBD3 and TCF4 constructs were chemically synthesized by ATCC and GeneArt (Thermofischer).

### Antibodies

pS63 c-Jun (#9261), pS73 c-Jun (#9164), JNK (#9252), pT183pY185 JNK (#9251), pT202pY204 ERK [20G11] (#4376), ERK [L34F12] (#4696), pT180pY182 p38 (#9211), p38 (#9212), and TCF4/TCF7l2 [C48H11] (#2569) from Cell Signalling; pT91 c-Jun [EPR2236] (#ab247509), pT93 c-Jun (#ab28854), and MBD3 [EPR9913] (#ab157464) from Abcam; c-Jun [3/Jun] (#610326) from BD Biosciences; Vinculin [hVIN-1] (#V9131), GST (#G7781) and Flag [M2] (#F3165) from Sigma-Aldrich; Peroxidase AffiniPure Goat anti-Mouse IgG (H + L) (#115-035-146), Peroxidase AffiniPure Goat anti-Rabbit IgG (H + L) (#111-035-144) (Jackson ImmunoResearch). Primary antibodies were used at 1:1000 dilution, except for Vinculin that was used 1:10000 and GST 1:20000. Secondary antibodies were used at 1:10000 dilution.

### Expression and purification of recombinant proteins

Wild-type or mutant human c-JUN sequences encoding residues 1-151 or full-length human MBD3 (residues 1-291) were inserted between the BamHI and NotI sites of modified pET-41a^13^ and expressed as GST-(His)_6_ fusion proteins. Protein expression was induced in *E. coli* Rosetta(DE3) (Novagen) with 0.5 mM IPTG for 3 h at 37 °C for c-JUN TAD, and in *E. coli* BL21(DE3) (Novagen) with 0.5 mM IPTG overnight at 18 °C for MBD3. Cells expressing c-JUN TAD or MBD3 were lysed in 50 mM Tris pH 8.0, 300 mM NaCl, 5 mM DTT, 5 mM MgCl_2_, 1 mM PMSF supplemented with EDTA-free protease inhibitor cocktail (Merck) and traces of Deoxyribonuclease I from bovine pancreas (Sigma). Cleared lysate was applied to glutathione sepharose resin (Cytiva), incubated for 1 h with the resin, and then washed extensively with lysis buffer. C-JUN proteins were either eluted with 20 mM reduced glutathione in 50 mM Tris pH 8.0, 150 mM NaCl, 5 mM DTT, or after adsorption to glutathione sepharose the c-JUN TAD proteins or MBD3 were released by incubation with 1:50 (w/w) GST-3C protease on the resin overnight at 4 °C, and further purified on a Superdex 200 HR10/30 size exclusion column (Cytiva) in 10 mM K_2_HPO_4_ pH 6.8, 50 mM NaCl for c-JUN TAD or in 25 mM Hepes pH 7.5, 100 mM NaCl, 5 mM DTT, 10% Glycerol for MBD3. For ^15^N- and ^15^N-/^13^C-labelling, cells were grown in M9 minimal medium containing ^15^NH_4_Cl and ^13^C-glucose as required and c-JUN was expressed for 3 h at 37 °C after induction with 0.5 mM IPTG. Human full-length TCF4 (residues 1-619) was inserted between the NcoI and EcoRI sites of a modified pET-30a vector (kind gift form Dr. Phil Robinson) carrying an N-terminal 6xHis tag and a TEV protease cleavage site. Protein expression was induced in *E. coli* BL21(DE3) (Novagen) with 0.5 mM IPTG overnight at 18 °C. Cells were lysed in 50 mM Tris pH 7.5, 300 mM NaCl, 2 mM β-mercaptoethanol, 5 mM MgCl_2_ and 5% glycerol, supplemented with 0.25 mg/ml lysozyme, EDTA-free protease inhibitor cocktail (Merck) and traces of Deoxyribonuclease I from bovine pancreas (Sigma), using an EmulsiFlex-C3 homogeniser (Avestin). Cleared lysate was loaded onto a 5 ml HisTrap column (Cytiva) equilibrated in the lysis buffer using an AKTA purifier (Cytiva). After extensive washing, the protein was eluted using an imidazole gradient 10–400 mM. The fractions containing purified TCF4, as confirmed by western blot analysis, were combined and imidazole was removed by dialysis.

### NMR spectroscopy

NMR data for assignment of c-JUN backbone resonances were acquired at 283 K using a Bruker Avance III NMR spectrometer with TXI cryoprobe operating at 700 MHz (^1^H Larmor frequency). A 150 μM sample of uniformly ^15^N,^13^C-labelled sample of c-JUN was prepared in 10 mM K_2_HPO_4_ pH 6.8, 150 mM NaCl, 5 mM MgCl_2_, 10% (v/v) D_2_O and 0.01% (w/v) DSS. BEST-HNCO, HNCACO, HNCACB and HNCOCACB experiments^44^ were acquired, processed using nmrPipe^45^ and analysed using CCPN Analysis (v2.5.1)^46^. Secondary structure populations were calculated from HN, N, CO, CA and CB chemical shifts using δ2D^28^. c-JUN phosphorylation kinetics were measured at 20 °C using a Bruker Avance III NMR spectrometer with TXI cryoprobe operating at 950 MHz (^1^H Larmor frequency) and running Topspin 3.5pl6. 391 μL samples of ^15^N-labelled cJun (WT or variants) were prepared in Shigemi tubes without plungers, at concentrations of ca. 75 μM in 10 mM K_2_HPO_4_ pH 6.8, 50 mM NaCl, 5 mM MgCl_2_, 2 mM DTT, 1 mM ATP, 5% (v/v) D_2_O. Reference 2D 1H,15N SOFAST-HMQC experiments^47^ were acquired using a 50 ms recycle delay, with 1536 complex points and a sweep width of 16 ppm in the direct dimension (100 ms acquisition time), and 64 complex points and a sweep width of 20 ppm in the indirect dimension (33.2 ms acquisition time). Phosphorylation was then initiated by rapid mixing with 290 U recombinant active JNK1 or JNK2 (Proqinase), and a series of SOFAST-HMQC experiments were acquired over a period of several hours, with 4 scans per increment (corresponding to 93 s per spectrum). This kinase concentration was optimised following initial estimates based on the specific activity, substrate concentration and the number of phosphorylation sites^23,24^ in order to obtain measurable levels of phosphorylation within hours, but not so fast that kinetics could not accurately be resolved by NMR. Spectra were processed in nmrPipe (v10.9)^45^ using linear prediction and cosine-squared window functions, then imported into Julia (v1.6) for analysis with NMRTools.jl (v0.0.1). Following previous studies^13,23,24^ box integrals were calculated around phosphorylated resonances and fitted to single exponential functions as a function of time. Phosphorylation progress was reported by normalising phosphorylated peak integrals according to the average of several well-resolved resonances (coefficient of variation (standard deviation/mean) of ca. 20%) in the N- and C-termini of the sequence, located far from phosphorylation sites.

### Cell culture

NIH3T3 and HCT116 were provided by the Francis Crick Institute Cell Services and cultured in Dulbeccos’s Modified Eagle Medium (DMEM) supplemented with 10% fetal bovine serum (FBS). Cell lines were tested for mycoplasma and authenticated by short-tandem repeat DNA profiling by the Francis Crick Institute Cell Services. Cells were treated when indicated with 50 µM JNK inhibitor (JNKi) (SP600125, Calbiochem) or 15 ng/ml anisomycin (Sigma). HCT116 cells were plated at subconfluence and transfected with lipofectamine 2000 (Invitrogen) when indicated.

### siRNA interference experiments

To silence MBD3 or TCF4, ON-TARGETplus MBD3 (Horizon Discovery, LQ-013616-00-0005) or TCF4/TCF7L2 (Horizon Discovery, LQ-003816-00-0005) siRNAs and a non-targeting control (Horizon Discovery, D-001810-01-05) were used. Cells were reversed-transfected with 25 nM small interfering RNAs (siRNAs) using Lipofectamine RNAiMAX (Invitrogen) for 48–72 h. Where indicated, cells were further treated with JNK inhibitor or anisomycin for 30 min.

### Phosphorylation assays

For phosphorylation of GST-c-JUN TAD, or the ^15^N isotope-labeled protein version, 75 μM of substrate was incubated with 290 U recombinant active JNK1 (Proqinase) in 10 mM K_2_HPO_4_ pH 6.8, 50 mM NaCl, 1 mM ATP, 2 mM DTT, 5 mM MgCl_2_ at 20 °C for the indicated times. For phosphorylation of endogenous JNK and c-JUN, cells were treated with 50 μM JNKi for 2 h followed by 15 ng/ml anisomycin treatment for the indicated times at 37 °C. Lysates for SDS-PAGE were prepared in 300 μl RIPA buffer (Thermo Fisher) supplemented with protease inhibitor (Sigma) and phosphatase inhibitor (Cell Signalling). Protein quantification was performed with the BCA kit (Thermo Scientific) and densitometry analysis of the immunoblot with the Image Studio Lite Software (Licor).

### Reporter gene assay

NIH3T3 cells were transiently transfected with pFA2-c-JUN WT (PathDetect c-JUN *trans*-Reporting System from Agilent Technologies), or the indicated pFA2-c-JUN mutants or pFC2-dbd (empty vector); pFR-Luc; pFC-MEKK; and tk-Renilla, using Lipofectamine 2000 (Invitrogen). Next day, medium was replaced with starvation medium (0.3% FBS) for 24 h. Luminescence activity was measured 48 h post-transfection using Dual-Luciferase Reporter Assay System following manufacturer’s protocol (Promega) in a FLUOStar Omega Plate reader (BMG Labtech). Data were expressed as fold induction after being normalized using tk-renilla luciferase.

### RNA isolation and quantitative PCR (RT-qPCR)

RNA was extracted using the RNeasy Mini Kit (Qiagen) according to the manufacturer’s instructions. One microgram of total RNA was reverse-transcribed using the iScript cDNA synthesis kit (BioRad) according to the manufacturer’s instructions. cDNA was diluted five-fold and 1 μL was used per RT-qPCR reaction. PowerUp SYBRGreen (Applied Biosystems) was used for real-time quantitative PCR (qPCR). Assays were performed on an ABI Applied Biosystems 7500 or QuantStudio Real-Time PCR System. RT-qPCR primers are listed in Supplementary Table 1.

### Immunoprecipitation

HCT116 cells were transiently transfected with pCMV-Tag2-Flag-cJUN (provided by Axel Behrens) wild-type or mutants, and were treated with JNKi or additionaly with anisomycin. Cells were lysed in Cell Lysis Buffer (Cell Signalling) with protease inhibitor (Sigma), phosphatase inhibitor (Cell Signalling) and 1 mM PMSF, and cleared by centrifugation for 15 min at 10,000 × *g* at 4 °C. Lysates were incubated with 20 μl of anti-FLAG M2 magnetic beads (Sigma) for 16 h at 4 °C. Beads were washed 3 times with Cell Lysis Buffer and proteins were eluted with 20 μl of 2x SDS loading buffer or with 3X FLAG peptide (Sigma).

### GST-fusion pulldown assays

HCT116 cells were lysed in Cell Lysis Buffer (Cell Signalling) with protease inhibitor (Sigma), phosphatase inhibitor (Cell Signalling) and 1 mM PMSF, sonicated and cleared by centrifugation for 15 min at 10,000 × *g* at 4 °C. 10 μl of gluthatione sepharose beads (Cytiva) were incubated with 10 μg GST c-JUN TAD fusion protein for 1 h at 4 °C. After binding beads were washed 3 times with lysis buffer, or 25 mM Hepes pH 7.5, 100 mM NaCL, 5 mM DTT, 10% Glycerol (for recombinant purified protein pulldowns) and were next incubated with the lysate or purified recombinant protein for 3 h at 4 °C. Beads were washed 3 times with Wash Buffer (50 mM Tris pH 7.5, 250 mM NaCl, 0.5% NP40, 5 mM EDTA and 1 mM DTT) (for endogenous MBD3) or Cell Lysis Buffer (for endogenous TCF4) or 25 mM Hepes pH 7.5, 100 mM NaCL, 5 mM DTT, 10% Glycerol for recombinant MBD3 and TCF4. Proteins were eluted with 20 μl of 1x SDS loading buffer.

### Statistics and reproducibility

Results are represented as mean or mean ± standard error of the mean (SEM) for bar graphs and scatter plots. Statistical significance was determined as described in the figure legends and calculated using GraphPad Prism 8. Briefly, one-way analysis of variance followed by Dunnett’s or Tukey’s correction for multiple comparisons was used. *p*-values are provided in the figure panels. A *p*-value of <0.05 was regarded as statistically significant for all datasets. The exact sample sizes (n) used to calculate statistics are provided in the figure legends. All biochemical and cell-based experiments were reproduced with similar results a minimum of three times. Time-resolved NMR measurements were performed on at least two biological independent replicates (*n* ≥ 2). Immunoblots images are representative of a minimum of three biologically independent experiments and uncropped scans of all representative immunoblots contained in the manuscript are shown in the Source Data file.

### Reporting summary

Further information on research design is available in the Nature Research Reporting Summary linked to this article.

## Supplementary information

Supplementary Information

Reporting Summary

## Data Availability

The data supporting the findings from this study are available within the manuscript and its supplementary information. The NMR data generated in this study have been deposited in the BMRB database under accession code 51638. The publicly available PDB structures 2XRW and 2WO6 were used in this study. Source data are provided with this paper.

## Source data

Source Data
