# Supplementary material for: An intrinsic temporal order of c-JUN N-terminal phosphorylation regulates its activity by orchestrating co-factor recruitment
Source: Nat Commun. 2022 Oct 17;13:6133. doi: 10.1038/s41467-022-33866-w (PMC9576782; doi:10.1038/s41467-022-33866-w)
Supplement: Supplementary file 1 — Supplementary Information [file 41467_2022_33866_MOESM1_ESM.pdf]

Supplementary Information

An intrinsic temporal order of c-JUN N-terminal phosphorylation regulates its activity by orchestrating co-factor recruitment

Christopher A. Waudby[1,2,10], Saul Alvarez-Teijeiro[3,4,10], E. Josue Ruiz[5,10], Simon Suppinger[3,6], Nikos Pinotsis[3], Paul R. Brown[7], Axel Behrens[5,8,9], John Christodoulou[1,3*], and Anastasia Mylona[3,8*]

[1]Institute of Structural and Molecular Biology, University College London, U.K.
[2]School of Pharmacy, University College London, U.K.
[3]Institute of Structural and Molecular Biology, Birkbeck College, University of London, U.K.
[4]Present address: Instituto de Investigación Sanitaria del Principado de Asturias (ISPA), Asturias, Spain; CIBERONC, Instituto de Salud Carlos III, Madrid, Spain
[5]Cancer Stem Cell Laboratory, Institute of Cancer Research, London, U.K.
[6]Present address: Friedrich Miescher Institute for Biomedical Research (FMI), Basel, Switzerland
[7]Randall Division of Cell and Molecular Biophysics, Guy's Campus, King's College, London, U.K.
[8]Division of Cancer, Department of Surgery and Cancer, Imperial College, London, U.K.
[9]Convergence Science Centre, Imperial College, London, SW7 2BU, U.K.

[10] These authors contributed equally: Christopher A. Waudby, Saul Alvarez-Teijeiro, E. Josue Ruiz

*Correspondence: j.christodoulou@ucl.ac.uk, amylona@ic.ac.uk

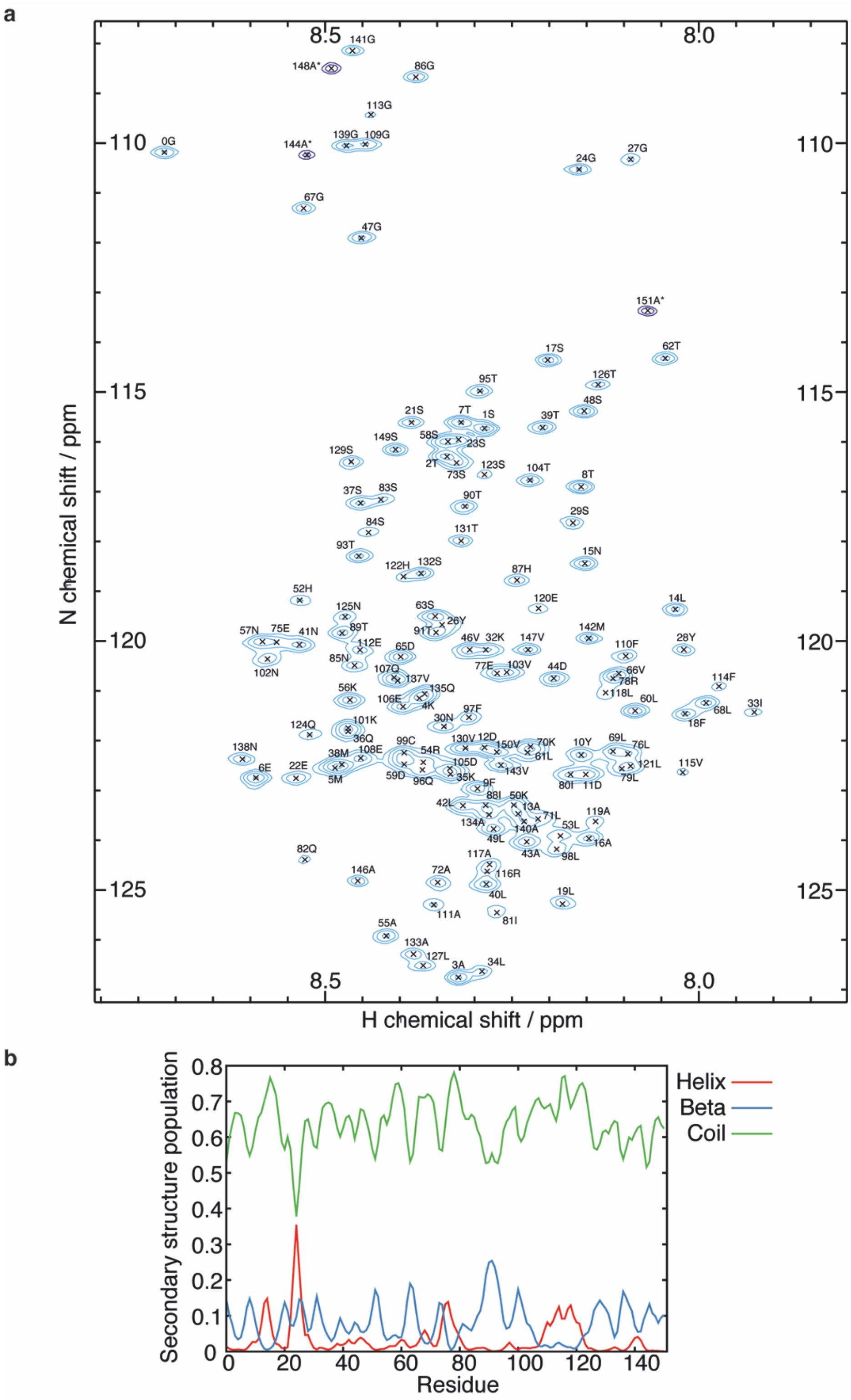

Supplementary Fig. 1. (**a**) ^{1}H,^{15}N HSQC spectrum of the c-JUN TAD (283 K, 700 MHz), showing resonance assignments. Assignments marked with an asterisk indicate folded resonances. (**b**) Secondary structure populations within the c-JUN TAD determined from HN, N, C', CA and CB chemical shifts using δ2D [28]. Source data are provided as a Source Data file.

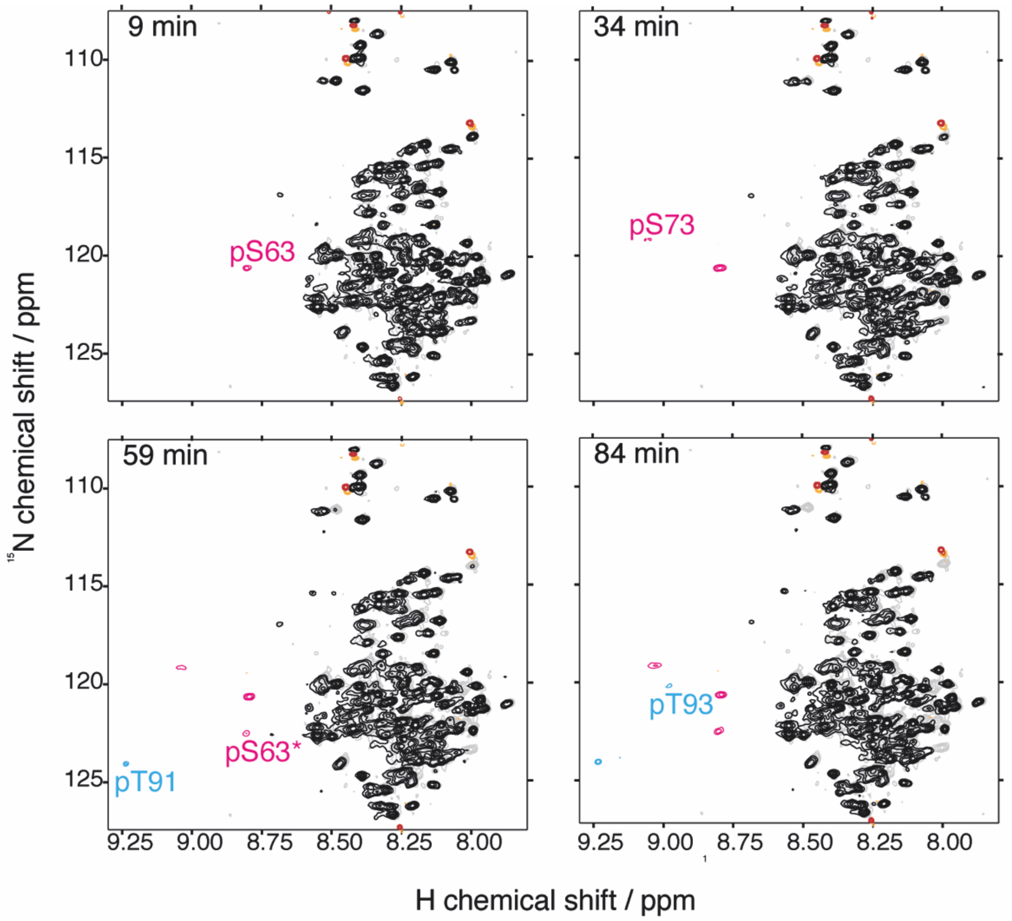

Supplementary Fig. 2. ^{1}H,^{15}N SOFAST-HMQC spectra of WT c-JUN TAD (293 K, 950 MHz) acquired at the indicated times following addition of active JNK1 to initiate phosphorylation. A spectrum of unphosphorylated c-JUN TAD is shown in grey for reference. Phosphorylated resonances have been highlighted in pink and cyan according to the kinetic group (fast / slow) with assignments as indicated. We note that two well-separated resonances were observed for pS63. In all analyses, we use the sum of these resonances as a reporter of pS63.

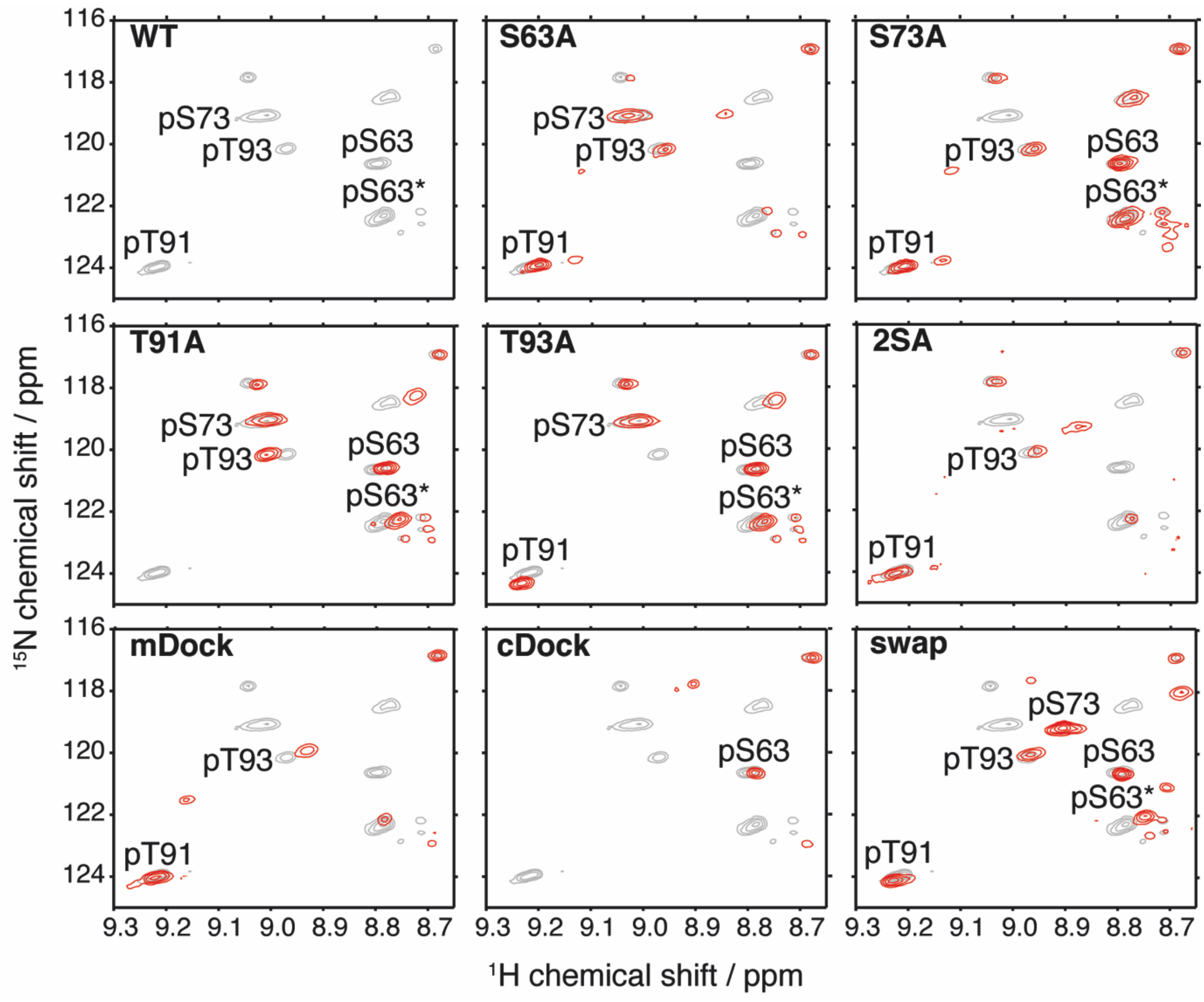

Supplementary Fig. 3. Assignment of phosphorylated c-JUN TAD amide resonances. Contour plots indicate the maximum values observed in $^{1}H,^{15}N$ SOFAST-HMQC spectra (293 K, 950 MHz) across a phosphorylation time course, for wild-type (WT) c-JUN TAD (grey) and variants as indicated (red). Resonance assignments are indicated on each panel.

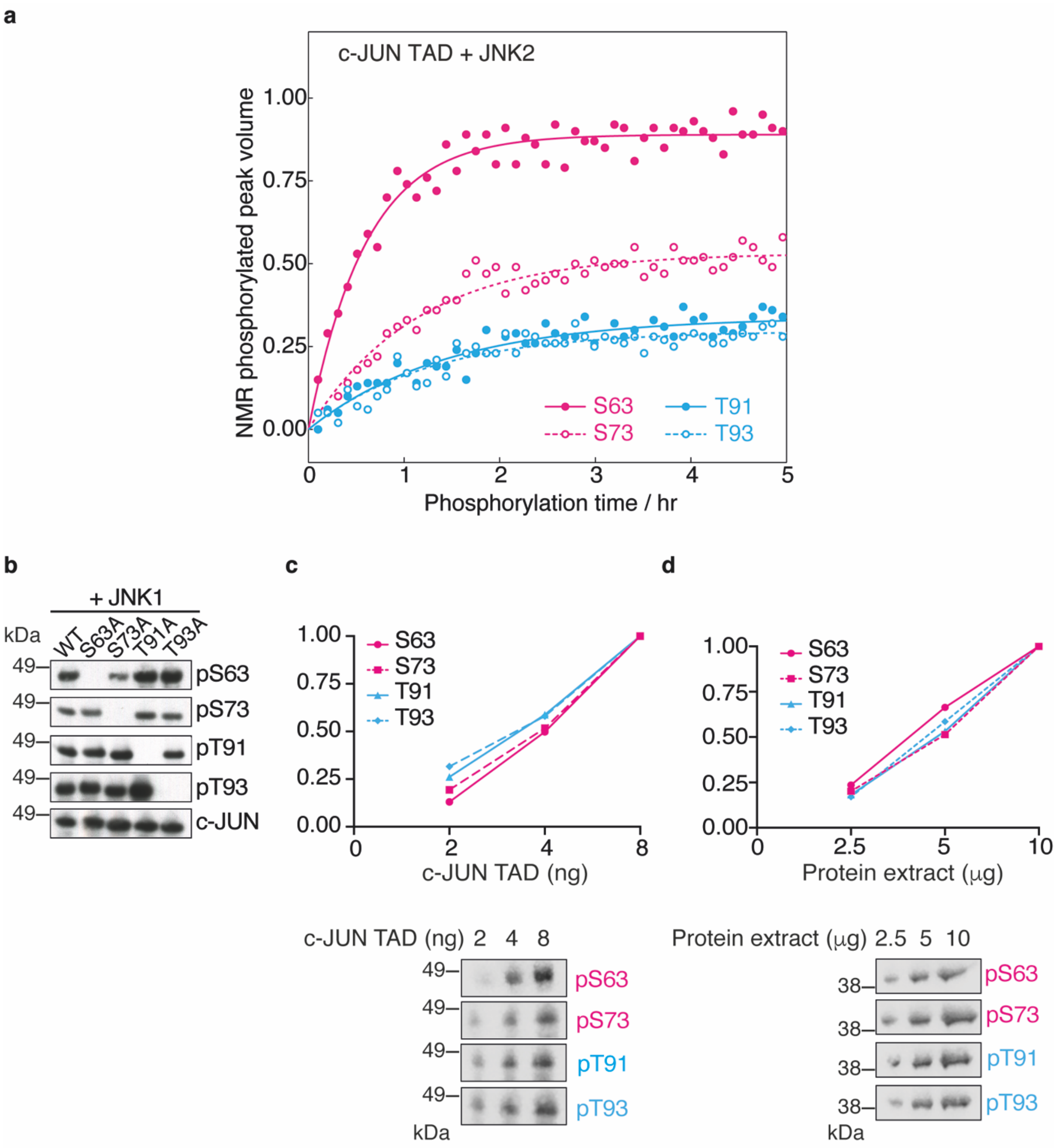

Supplementary Fig. 4. (**a**) Time-resolved modification curves of wild-type c-JUN TAD individual sites upon phosphorylation with active JNK2. (**b**) Phospho-specific immunoblotting analysis of recombinant in vitro phosphorylated wild-type and alanine point mutants (S63A, S73A, T91A, T93A) c-JUN TAD. (**c** and **d**) Linear range of detection for each c-JUN phosphorylation-specific antibody was validated by serial dilutions of the saturated phosphorylation kinetics time point of purified GST-c-JUN TAD (Fig. 1d) (**c**) or endogenous c-JUN (Fig. 2b) (**d**). All immunoblots in panels b-d are representative of three biologically independent (n = 3) experiments obtained with similar results. Source data are provided as a Source Data file.

a

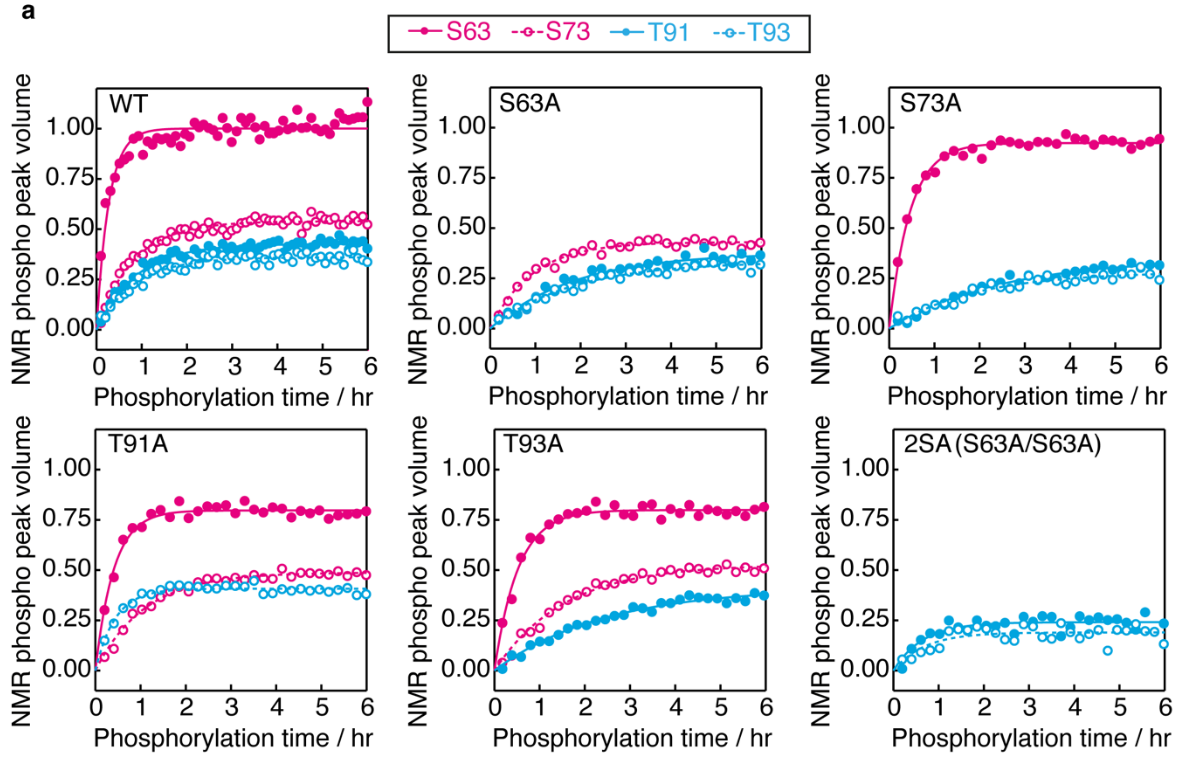

b

WT c-JUN TAD
```
1-MTAKMETTFYDDALNASFLPSESGPYGYSNPKILKQSMTLNLADPVGSLKPHLRA
  KNSDLLTSPDVGLLKLASPELERLIIQSSNGHITTTPTPTQFLCPKNVTDEQEGF
  AEGFVRALAELHSQNTLPSVTSAAQPVNGAGMVAPAVASVA-151
```

mDOCK c-JUN TAD
```
1-MTAKMETTFYDDALNASFLPSESGPYGYSNPPHLRAKNSDLLTSPDVGLLKLASP
  ELERLIIQKILKQSMTLNLADPVGSLKSSNGHITTTPTPTQFLCPKNVTDEQEGF
  AEGFVRALAELHSQNTLPSVTSAAQPVNGAGMVAPAVASVA-151
```

cDOCK c-JUN TAD
```
1-MTAKMETTFYDDALNASFLPSESGPYGYSNPPHLRAKNSDLLTSPDVGLLKLASP
  ELERLIIQSSNGHITTTPTPTQFLCPKNKILKQSMTLNLADPVGSLKVTDEQEGF
  AEGFVRALAELHSQNTLPSVTSAAQPVNGAGMVAPAVASVA-151
```

c

WT c-JUN TAD (63, 73, 91 93)
```
N -NSDLLTSPDVGLLKLASPELERL-/-NGHITTTPTPTQFLC- C
```

swap c-JUN TAD (91 93, 63, 73)
```
N -NGHITTTPTPTQFLC-/-NSDLLTSPDVGLLKLASPELERL- C
```

TTSS c-JUN TAD
```
1-MTAKMETTFYDDALNASFLPSESGPYGYSNPKILKQSMTLNLADPVGSLKPHLRA
  KNSDLLTTPDVGLLKLATPELERLIIQSSNGHITTSPSPTQFLCPKNVTDEQEGF
  AEGFVRALAELHSQNTLPSVTSAAQPVNGAGMVAPAVASVA-151
```

Supplementary Fig. 5. (**a**) Time-resolved modification curves of individual sites upon phosphorylation with JNK1 for S63A, S73A, T91A, T93A and 2SA variants comparing to wild-type (WT). Source data are provided as a Source Data file. (**b**) Amino acid sequence changes of the mDock and cDock c-JUN TAD constructs analysed by time-resolved NMR, comparing to the WT sequence. (**c**) Amino acid sequence changes of the swap and TTSS c-JUN TAD constructs analysed by time-resolved NMR, comparing to the WT sequence.

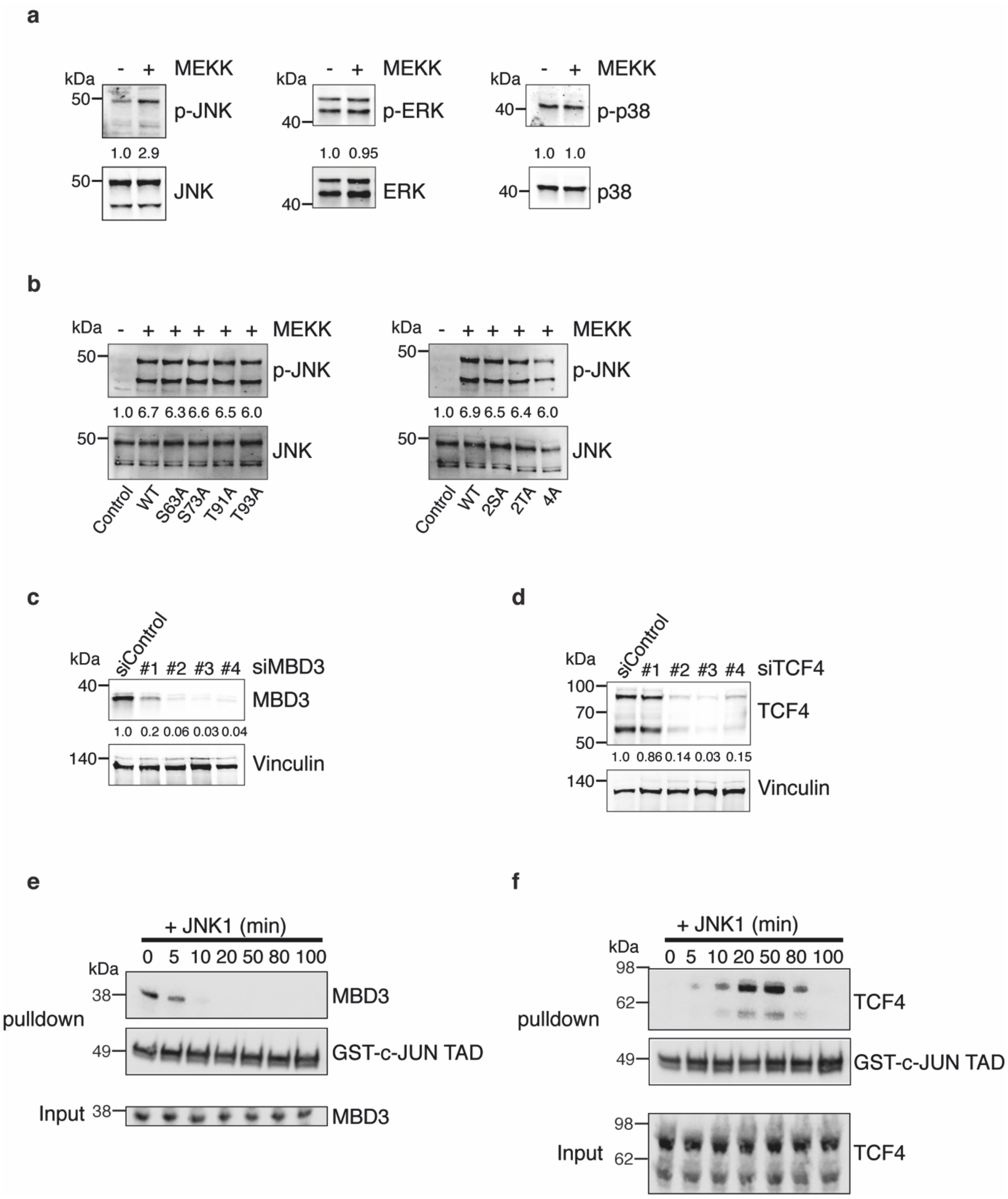

Supplementary Fig. 6. (**a**) NIH3T3 cells were transiently transfected to express a constitutively active MEKK (pFC-MEKK) as an upstream activator of JNK, (**b**) together with c-JUN wild-type (pFA2-c-JUN WT) or c-JUN alanine mutants (Left; pFA2-c-JUN S63A, pFA2-c-JUN S73A, pFA2-c-JUN T91A or pFA2-c-JUN T93A. Right; pFA2-c-JUN 2SA, pFA2-c-JUN 2TA or pFA2-c-JUN 4A). (**c** and **d**) siRNA-mediated knockdown of MDB3 or TCF4 in HCT116 human colorectal cancer cells. Vinculin is shown as loading control. siRNA #3 showed the best knockdown and cell survival for both MBD3 and TCF4 and thus used for the described experiments. (**e** and **f**) HCT116 cell extracts were used in pulldown assays, using

GST-c-JUN TAD as bait, phosphorylated by JNK1 at the indicated times. Recovered proteins were analysed by immunoblotting for MBD3 (**e**) or TCF4 (**f**). A long and a short TCF4 isoform is recognised by the antibody used and both could be recovered in this assay. All immunoblots in panels a-f are representative of three biologically independent (n = 3) experiments obtained with similar results. Source data are provided as a Source Data file.

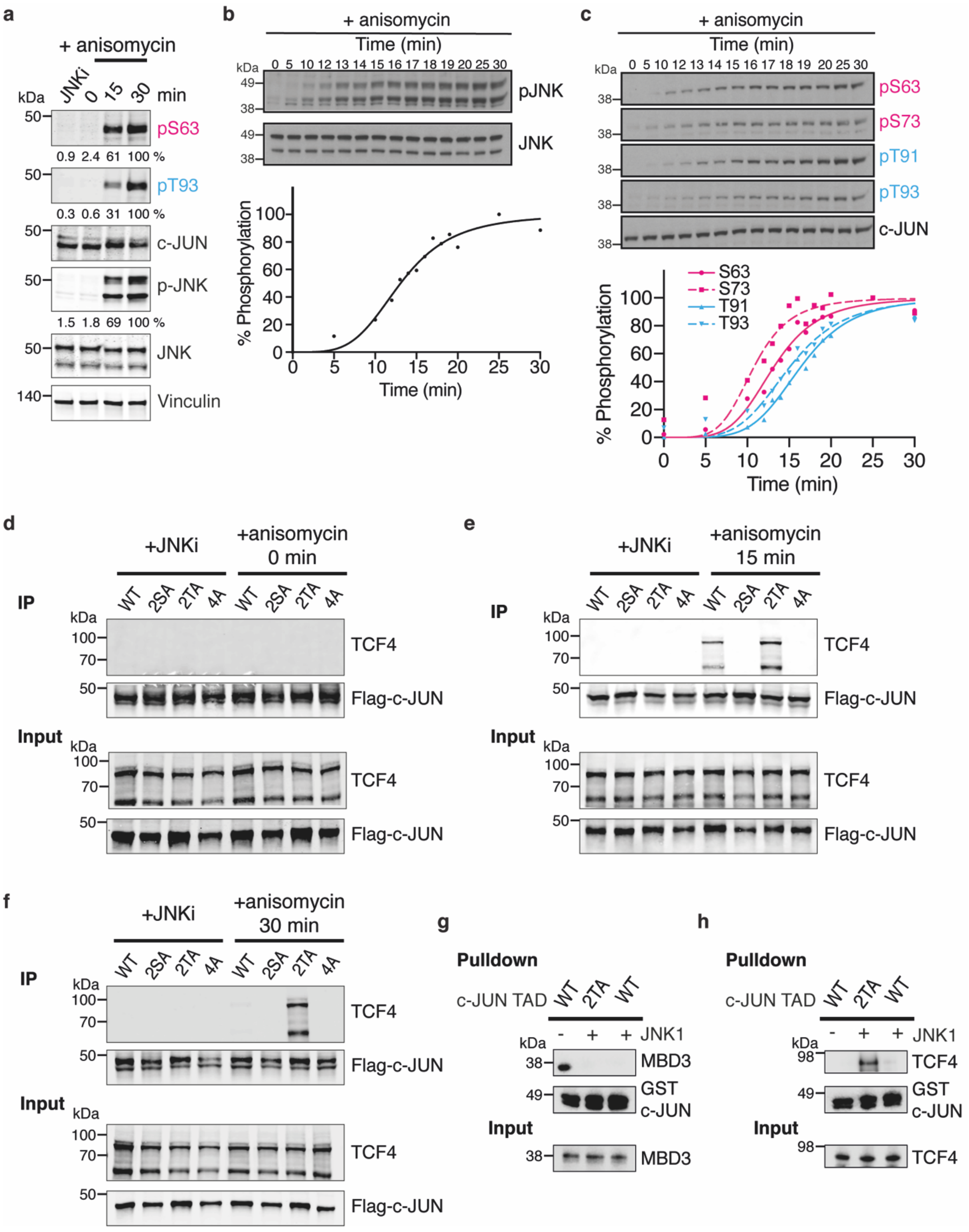

Supplementary Fig. 7. (**a**) Time-course immunoblot analysis of endogenous JNK and c-JUN phosphorylation in HCT116 cells following anisomycin treatment for the indicated times. (**b**) Time-course immunoblot analysis of endogenous JNK phosphorylation/activation in HCT116 cells following anisomycin treatment for the indicated times (top), and quantification of the detected protein levels by western blot using Image Studio Lite Software (bottom). (**c**) Phospho-specific immunoblotting analysis of endogenous c-JUN time-course phosphorylation

in HCT116 cells following anisomycin treatment for the indicated times (top), and quantification of the detected protein levels by western blot using Image Studio Lite Software (bottom). (**d, e** and **f**) The indicated flag-tagged c-JUN wild-type or derivatives, transiently overexpressed in HCT116 cells, treated with either JNKi or stimulated additionally with anisomycin for 0, 15 or 30 min, were immunoprecipitated using Flag antibody. Immunoprecipitates were analysed for interaction with endogenous TCF4. Both the long and short TCF4 isoform could be recovered. (**g** and **h**) Recombinant unphosphorylated or fully phosphorylated GST wild-type c-JUN TAD or GST 2TA TAD derivative was used as bait in pulldowns with recombinant MBD3 (**g**) and TCF4 (**h**). Recovered proteins were analysed by immunoblotting for MBD3 (**g**) and TCF4 (**h**). All immunoblots in panels a-h are representative of three biologically independent (n = 3) experiments obtained with similar results. Source data are provided as a Source Data file.

Supplementary Table 1.
Primers for quantitative polymerase chain reaction (qPCR).

Name	Primer (5′–3′)	
	Forward	Reverse
ACTIN	GAAAATCTGGCACCACACCT	TAGCACAGCCTGGATAGCAA
c-JUN	TCCAAGTGCCGAAAAAGGAAG	CGAGTTCTGAGCTTTCAAGGT
