# Supplementary material for: An intrinsic temporal order of c-JUN N-terminal phosphorylation regulates its activity by orchestrating co-factor recruitment
Source: Nat Commun. 2022 Oct 17;13:6133. doi: 10.1038/s41467-022-33866-w (PMC9576782; doi:10.1038/s41467-022-33866-w)
Supplement: Supplementary file 2 — Reporting Summary [file 41467_2022_33866_MOESM2_ESM.pdf]

nature portfolio

Corresponding author(s): Anastasia Mylona, John Christodoulou

Last updated by author(s): Sep 20, 2022

Reporting Summary

Nature Portfolio wishes to improve the reproducibility of the work that we publish. This form provides structure for consistency and transparency in reporting. For further information on Nature Portfolio policies, see our Editorial Policies and the Editorial Policy Checklist.

Statistics

For all statistical analyses, confirm that the following items are present in the figure legend, table legend, main text, or Methods section.

n/a	Confirmed	
☐	☒	The exact sample size (*n*) for each experimental group/condition, given as a discrete number and unit of measurement
☐	☒	A statement on whether measurements were taken from distinct samples or whether the same sample was measured repeatedly
☐	☒	The statistical test(s) used AND whether they are one- or two-sided<br>*Only common tests should be described solely by name; describe more complex techniques in the Methods section.*
☒	☐	A description of all covariates tested
☐	☒	A description of any assumptions or corrections, such as tests of normality and adjustment for multiple comparisons
☐	☒	A full description of the statistical parameters including central tendency (e.g. means) or other basic estimates (e.g. regression coefficient) AND variation (e.g. standard deviation) or associated estimates of uncertainty (e.g. confidence intervals)
☐	☒	For null hypothesis testing, the test statistic (e.g. *F*, *t*, *r*) with confidence intervals, effect sizes, degrees of freedom and *P* value noted<br>*Give P values as exact values whenever suitable.*
☒	☐	For Bayesian analysis, information on the choice of priors and Markov chain Monte Carlo settings
☒	☐	For hierarchical and complex designs, identification of the appropriate level for tests and full reporting of outcomes
☒	☐	Estimates of effect sizes (e.g. Cohen's *d*, Pearson's *r*), indicating how they were calculated

Our web collection on statistics for biologists contains articles on many of the points above.

Software and code

Policy information about availability of computer code

Data collection	NMR data:Bruker Topspin 3.5pl6; Quantitative PCR: 7500 fast and QuantStudio 7; Image Studio Lite 5.2.5. Software
Data analysis	NMR data: CCPN Analysis 2.5.1; nmrPipe 10.9 (Delaglio F, Grzesiek S, Vuister GW, Zhu G, Pfeifer J, Bax A. NMRPipe: a multidimensional spectral processing system based on UNIX pipes. J Biomol NMR. 1995;6: 277–293.); Julia 1.6 (Bezanson J, Edelman A, Karpinski S, Shah VB. Julia: A fresh approach to numerical computing. SIAM Rev Soc Ind Appl Math. 2017;59: 65–98.); NMRTools.jl 0.0.1 (Waudby C, 2021, doi:10.5281/zenodo.5585089); Other biochemical data: ImageStudioLite 5.2.5; GraphPad Prism 8

For manuscripts utilizing custom algorithms or software that are central to the research but not yet described in published literature, software must be made available to editors and reviewers. We strongly encourage code deposition in a community repository (e.g. GitHub). See the Nature Portfolio guidelines for submitting code & software for further information.

Data

Policy information about availability of data

All manuscripts must include a data availability statement. This statement should provide the following information, where applicable:

- Accession codes, unique identifiers, or web links for publicly available datasets
- A description of any restrictions on data availability
- For clinical datasets or third party data, please ensure that the statement adheres to our policy

The data supporting the findings from this study are available within the manuscript and its supplementary information. The NMR data generated in this study have been deposited in the BMRB database under accession code 51638 [www.bmrb.io]. The PDB 2XRW [10.2210/pdb2XRW/pdb] and 2WO6 [10.2210/pdb2WO6/pdb]

structures were used in this study. Source data are provided with this paper.

Human research participants

Policy information about studies involving human research participants and Sex and Gender in Research.

Reporting on sex and gender	N/A
Population characteristics	N/A
Recruitment	N/A
Ethics oversight	N/A

Note that full information on the approval of the study protocol must also be provided in the manuscript.

Field-specific reporting

Please select the one below that is the best fit for your research. If you are not sure, read the appropriate sections before making your selection.

[x] Life sciences [ ] Behavioural & social sciences [ ] Ecological, evolutionary & environmental sciences

For a reference copy of the document with all sections, see nature.com/documents/nr-reporting-summary-flat.pdf

Life sciences study design

All studies must disclose on these points even when the disclosure is negative.

Sample size	No statistical methods were used to predetermine sample size. All NMR measurements included at least two independent biological replicates under the same conditions and with same batch of kinase, as typical for time-resolved NMR studies. All samples undergo rigorous biochemical and NMR quality measurements, as described. All other experiments included at least 3 independent biological replicates to ensure enough power to draw conclusions based on a standard α cut-off of 0.05 in statistical tests.
Data exclusions	No data were excluded from analysis.
Replication	All experiments were repeated multiple times and/or on multiple biological replicates with similar results as indicated in the figure legends.
Randomization	Randomization was not performed because the biochemical analysis was done according to treatment, and also it is not typical for NMR studies.
Blinding	Blinding was not applicable in the NMR and other biochemical assays in this manuscript. The data are based on quantitative analysis and are not subjective.

Reporting for specific materials, systems and methods

We require information from authors about some types of materials, experimental systems and methods used in many studies. Here, indicate whether each material, system or method listed is relevant to your study. If you are not sure if a list item applies to your research, read the appropriate section before selecting a response.

Materials & experimental systems

n/a	Involved in the study	
[ ]	[x]	Antibodies
[ ]	[x]	Eukaryotic cell lines
[x]	[ ]	Palaeontology and archaeology
[x]	[ ]	Animals and other organisms
[x]	[ ]	Clinical data
[x]	[ ]	Dual use research of concern

Methods

n/a	Involved in the study	
[x]	[ ]	ChIP-seq
[x]	[ ]	Flow cytometry
[x]	[ ]	MRI-based neuroimaging

Antibodies

Antibodies used	pS63 c-Jun (#9261), pS73 c-Jun (#9164), JNK (#9252), pT183pY185 JNK (#9251), pT202pY204 ERK [20G11] (#4376), ERK [L34F12] (#4696), pT180pY182 p38 (#9211), p38 (#9212), and TCF4/TCF7l2 [C48H11] (#2569) from Cell Signalling; pT91 c-Jun [EPR2236] (#ab247509), pT93 c-Jun (#ab28854), and MBD3 [EPR9913] (#ab157464) from Abcam; c-Jun [3/Jun] (#610326) from BD Biosciences;

	Vinculin [hVIN-1] (#V9131), GST (#G7781) and Flag [M2] (#F3165) from Sigma-Aldrich; Peroxidase AffiniPure Goat anti-Mouse IgG (H+L) (#115-035-146), Peroxidase AffiniPure Goat anti-Rabbit IgG (H+L) (#111-035-144) (Jackson ImmunoResearch). Primary antibodies were used at 1:1000 dilution, except for Vinculin that was used 1:10000 and GST 1:20000. Secondary antibodies were used at 1:10000 dilution.
Validation	All antibodies were validated for use in the assay and species under study by the suppliers and are commonly used for WB (see company websites).<br><br>pS63 c-Jun (#9261, Cell Signalling), https://www.cellsignal.co.uk/products/primary-antibodies/phospho-c-jun-ser63-ii-antibody/9261<br>pS73 c-Jun (#9164, Cell Signalling), https://www.cellsignal.co.uk/products/primary-antibodies/phospho-c-jun-ser73-antibody/9164<br>JNK (#9252, Cell Signalling), https://www.cellsignal.co.uk/products/primary-antibodies/sapk-jnk-antibody/9252<br>pT183pY185 JNK (#9251, Cell Signalling), https://www.cellsignal.co.uk/products/primary-antibodies/phospho-sapk-jnk-thr183-tyr185-antibody/9251<br>pT202pY204 ERK [20G11] (#4376, Cell Signalling), https://www.cellsignal.co.uk/products/primary-antibodies/phospho-p44-42-mapk-erk1-2-thr202-tyr204-20g11-rabbit-mab/4376<br>ERK [L34F12] (#4696, Cell Signalling), https://www.cellsignal.co.uk/products/primary-antibodies/p44-42-mapk-erk1-2-l34f12-mouse-mab/4696?_=1663168958396&Ntt=4696&tahead=true<br>pT180pY182 p38 (#9211, Cell Signalling), https://www.cellsignal.co.uk/products/primary-antibodies/phospho-p38-mapk-thr180-tyr182-antibody/9211<br>p38 (#9212, Cell Signalling), https://www.cellsignal.co.uk/products/primary-antibodies/p38-mapk-antibody/9212?_=1663168996146&Ntt=9212&tahead=true<br>TCF4/TCF7l2 [C48H11] (#2569, Cell Signalling) https://www.cellsignal.co.uk/products/primary-antibodies/tcf4-tcf7l2-c48h11-rabbit-mab/2569?site-search-type=Products&N=4294956287&Ntt=c48h11&fromPage=plp<br>pT91 c-Jun [EPR2236] (#ab247509, Abcam), https://www.abcam.com/c-jun-phospho-t91-antibody-epr2236-bsa-and-azide-free-ab247509.html<br>pT93 c-Jun (#ab28854, Abcam), http://www.abcam.com/c-jun-phospho-t93-antibody-ab28854.html<br>MBD3 [EPR9913] (#ab157464, Abcam) https://www.abcam.com/mbd3-antibody-epr9913-chip-grade-ab157464.html<br>c-Jun [3/Jun] (#610326, BD Biosciences), https://www.bdbiosciences.com/en-us/products/reagents/microscopy-imaging-reagents/immunofluorescence-reagents/purified-mouse-anti-jun.610326<br>Vinculin [hVIN-1] (#V9131, Sigma-Aldrich), https://www.sigmaaldrich.com/GB/en/product/sigma/v9131<br>GST (#G7781, Sigma-Aldrich), https://www.sigmaaldrich.com/GB/en/product/sigma/g7781<br>Flag [M2] (#F3165, Sigma-Aldrich); https://www.sigmaaldrich.com/GB/en/product/sigma/f3165<br>Peroxidase AffiniPure Goat anti-Mouse IgG (H+L) (#115-035-146, Jackson ImmunoResearch), https://www.jacksonimmuno.com/catalog/products/115-035-146<br>Peroxidase AffiniPure Goat anti-Rabbit IgG (H+L) (#111-035-144,Jackson ImmunoResearch), https://www.jacksonimmuno.com/catalog/products/111-035-144

Eukaryotic cell lines

Policy information about cell lines and Sex and Gender in Research

Cell line source(s)	Cell Services of the Francis Crick Institute for 3T3 and HCT116 cells.
Authentication	All cells lines were authenticated using STR profiling by the Cell Service of The Francis Crick Institute.
Mycoplasma contamination	All cell lines were obtained mycoplasma free from Cell Services of the Francis Crick Institute.
Commonly misidentified lines (See ICLAC register)	No commonly misidentified cell lines were used in this study.
